# Form-Focused Instruction, Technology, and Limitations: An Investigation of Past Research and Future Possibilities

**DOI:** 10.3389/fpsyg.2022.920818

**Published:** 2022-06-23

**Authors:** Andrew Schenck

**Affiliations:** Faculty of Sciences and Humanities, SUNY Korea, Seoul, South Korea

**Keywords:** form-focused instruction, processability theory, L1, grammar, corrective feedback (CF)

## Abstract

Modern studies that integrate technology with form-focused instruction (FFI) are useful, yet analysis of grammatical or learner differences is often limited. Without consistent examination of all factors that influence the efficacy of FFI, it is no surprise that results are often inconsistent. Recent synthesis of research concerning Korean, Persian, and Chinese learners suggests that grammatical difficulty (collectively defined by grammatical complexity, learner proficiency, and L1) determines when providing input is more effective, and when prompting a learner to produce output is more effective. Results suggest that input and explicit information may help learners produce language more accurately when grammatical difficulty is high (e.g., a grammatical feature is complex and learner proficiency is low). Conversely, compelling a learner to produce output appears to increase accuracy when difficulty of a grammatical feature is low (e.g., a grammatical feature is not complex and proficiency is high). More experimental research is needed to confirm results. Such research may provide a holistic perspective needed to tailor computer software to learner needs.

## Introduction

Instead of human-to-human interaction, modern studies of corrective feedback (CF) tend to focus mainly on the use of technology. One recent study of written CF, for example, examined how computer-mediated collaborative groupwork could impact acquisition of the English definite and indefinite articles. Results of the study suggested that there was a significant relationship between CF and learner accuracy over time (Yamashita, 2021). In another study of the English article, the effects of digital game-based instruction vs. teacher instruction with corrective feedback were examined in more detail. Results suggested that only teacher instruction was more effective on the immediate posttest; the game-based instruction group, however, was significantly different for the delayed posttest (Reynolds and Kao, 2019). In a recent study of video chat recasts vs. face-to-face recasts, 57 Persian learners were found to use English articles more accurately in an oral production task for video chat recasts, suggesting superiority over face-to-face delivery (Rassaei, 2017).

While the use of computer-based media for grammar emphasis is indeed an important issue to explore, modern research appears to have some key limitations that reflect inherent weaknesses in past experimental designs. Studies by Yamashita (2021); Reynolds and Kao (2019), and Rassaei (2017), for example, all examine the same grammatical feature. A tendency to use the same grammatical feature in experimental studies appears to reflect a past misconception, which proposed that there are only two types of grammar. Systematic features like the past regular tense or English article were thought to be treatable, whereas lexical features (vocabulary words or collocations) were considered non-treatable (Wang and Jiang, 2015). Using an overly simplistic classification of either rule-governed or lexical features has dominated the field of second language acquisition, explaining why studies like that of Shintani (2012) test just vocabulary (nouns and adjectives) and one grammatical morpheme (plural -*s*). In addition to overly simple classification of features according to rule-based or lexical qualities, past research has used overly simplistic categorizations like simple or complex and early or late (Varnosfadrani and Basturkmen, 2009; Spada and Tomita, 2010; Van De Guchte et al., 2015). In reality, characteristics of a grammatical feature vary considerably based upon semantic, morphosyntactic, and phonological characteristics, which appear to collectively influence how a target feature is acquired (Goldschneider and DeKeyser, 2005).

In addition to oversimplistic categorization of grammar, research often lacks important information about learner background (Liu and Brown, 2015). Concerning the study by Yamashita (2021), 48 ESL learners from a United States university were examined. While information about proficiency was provided, levels varied and five could not be reported, since they lacked a score from a standardized exam like the TOEFL. The scores obtained led the researcher to conclude that “the sample was approximately upper-intermediate to advanced” (p. 80). These learners also came from various L1 backgrounds. While the participants were mostly Chinese (*n* = 20), other learners with Arabic, Indonesian, and Japanese L1s were represented. Concerning the study by Reynolds and Kao (2019), learners were recruited through a call for participants advertised at four schools of management in northern Taiwan with “ability to write at least 200 words in one sitting without major difficulties” (p. 466). No other information about participants was provided, leading to questions about L1 and English proficiency, both of which are known determinants of acquisition. As for the study by Rassaei (2017), all of the 57 learners had Persian L1s. Learners were described as “studying English at level 7 of an English program with level 12 as the highest level” (p. 138). Although this does give us some understanding of proficiency, more specific standardized assessment could allow for generalization to other learners of similar ability levels.

## Examining the Multiple Factors of Acquisition

Before the significance of CF studies can be accurately assessed, multiple influences of acquisition must be examined and understood. First, grammar is more diverse than simple binary dichotomies would suggest, impacting the process of acquisition. According to the Processability Theory, for example, grammar varies based upon whether it modifies a single phrase (verb, noun, or adjective), multiple phrases, or larger clauses. More simplistic grammar that modifies a single phrase tends to be acquired first (e.g., plural -s, verb tenses, etc.). Next, grammar that expresses a relationship between multiple phrases begins to emerge (e.g., do support, subject/verb inversion, or phrasal verbs). Finally, larger clauses are used and manipulated at the sentence level (e.g., conditional sentences, relative clauses, or cancel inversion) (Pienemann and Lenzing, 2015).

Since some grammatical features are more difficult to use, learners must have a certain level of proficiency to acquire a grammatical feature. Research supports this assertion, suggesting that grammar given just above the level of an ESOL learner makes promotes acquisition (Dyson and Håkansson, 2017; Dyson, 2018). Other characteristics of the learner like L1 may influence the acquisition process, either serving to promote or hinder acquisition according to morphosyntactic similarities (Luk and Shirai, 2009; Shin, 2015; Yang et al., 2017). L1 processing routines have even been shown to be effective in promoting the acquisition of L2 grammar (McManus and Marsden, 2019).

A final influence on acquisition is the CF itself. Type of CF may influence how a grammatical feature is acquired. Reformulations, for example, provide a scaffold, since a correction is given. Prompts, in contrast, push the learner to correct their own error, making the process of acquisition more cognitively demanding. Explicit forms of CF, which overtly direct attention to a language form (e.g., all forms of written feedback) may also influence the efficacy of CF (Li and Vuono, 2019). Despite potential influences, past studies have not systematically examined each type of CF with a variety of grammatical features. Research by Lyster et al. (2013) goes further to explore different types of grammatical features with CF, yet it still conceptualizes target features with an overly simplistic dichotomy based on grammatical and lexical characteristics. Furthermore, discussion of treatment efficacy and grammatical feature type is based primarily on the target feature itself, lacking related information on learner background variables like proficiency, which may ultimately impact the difficulty level of a grammatical feature (and subsequent effectiveness of a treatment).

## A Need for a Holistic Perspective in Examination of Corrective Feedback

Without consistent examination of all factors that influence the efficacy of CF (grammatical complexity, proficiency, and L1), it is no surprise that results are often inconsistent. Some researchers, for example, argue that written CF is effective (Bitchener et al., 2005; Chen et al., 2016), whereas others suggest that it is largely unnecessary (Truscott, 1996, 1999). Some scholars contend that oral recasts are very useful (Sakai, 2011; Goo and Mackey, 2013; Banaruee et al., 2018), while others argue that “The acquisitional value of recasts in comparison to other forms of corrective feedback might have been overestimated” (Ellis and Sheen, 2006, p. 575). Past experimental studies have failed to provide a comprehensive understanding of the impact of CF, which is ultimately needed to adapt theory to practice.

To provide a more holistic perspective of multiple variables influencing the acquisition process, studies of CF were synthesized for learners with Korean, Persian, and Chinese L1s (Schenck, 2020, 2021, 2022b). Results suggest a potential pedagogical application of theory to practice. The effectiveness of different types of CF on accuracy of production (implicit knowledge in assessments of either writing or speaking) appeared to be impacted by grammatical difficulty, which was defined by the type of grammatical feature, along with learner characteristics of proficiency and L1. Concerning the past regular tense, for example, Korean learners who tend to be at lower levels of proficiency had average effect sizes that were larger for implicit reformulations, in the form of oral recasts (Schenck, 2020). While this information is insightful, differences in research methodology between the two studies averaged for analysis (Cho, 2012; Kim and Cho, 2017) need to be considered. In the study by Kim and Cho (2017), for example, only two thirds of the learners examined were at the beginner level, with the remainder being labeled intermediate. Furthermore, only the study by Cho (2012) contained treatments with both recasts and prompts; from this study, results revealed that improvements in accuracy due to recasts were either higher (for the past irregular tense) or nearly the same as the prompt (for the past regular -*ed*). Overall, average effect sizes for both regular and irregular past tenses were higher for recasts, which appears to suggest that such recasts are more effective when emphasizing the past tense at lower proficiency levels. While insightful, variability in research methodology of past experimental studies also reveals a need for further, more holistic experimental examination of FFI.

Both Chinese and Persian learners, all of whom were at intermediate levels of proficiency, benefited more from implicit oral prompts (Yang and Lyster, 2010; Khezrlou, 2019). These studies suggest that more simplistic, intra-phrasal features like the past tense require a scaffold (a correction) at beginner levels, whereas more cognitively demanding prompts are required at intermediate levels. In addition to this finding, research synthesis also suggests that lower-level learners benefit more from explicit CF, which promotes conscious consideration of the target feature (Schenck, 2022b). Overall, findings appear to suggest that recasts and explicit techniques serve as a form of scaffold earlier in the process of acquisition, helping lower-level learners produce the target feature more accurately. Prompts, in contrast, tend to be more effective with learners at higher levels, who may have sufficient knowledge of the target feature and may not need a scaffold (Table 1).

**TABLE 1:** Type of assistance provided by each form of corrected feedback.

Type of CF	Difficulty of grammatical feature	Degree of assistance
Implicit Prompt (Oral Prompt)	Lowest	Least Scaffolded
Explicit Prompt (Metalinguistic Written Feedback)		
Implicit Reformulation (Oral Recast)		
Explicit Reformulation (Direct Written Feedback)	Highest	Most Scaffolded

It appears that prompts are more effective when proficiency and competence with a feature increase, while scaffolded reformulations are more effective when proficiency is low and grammatical difficulty is high. Explicit FFI also appears to be more effective than implicit FFI, suggesting that this characteristic of CF also serves as a scaffold.

## Similar Patterns in Input-Based and Output-Based Instruction

As in the case of CF, results of other FFI techniques that emphasize either input or output have been relatively inconsistent. Some studies suggest that modification of input increases grammatical accuracy and noticing (Jourdenais et al., 1995; Lee, 2007), whereas other studies reveal that such modification is rather inconsequential (Leow et al., 2003; Lee and Huang, 2008). Several studies suggest that facilitating output helps learners to acquire grammatical features and vocabulary (Izumi, 2002; Shintani, 2011; Rassaei, 2012), yet other research implies that output-based activities do not adequately engage the learner (Izumi and Bigelow, 2000; Izumi and Izumi, 2004). As past studies reveal, the true efficacy of FFI techniques that emphasize either input or output has yet to be realized. As in the case of CF, more holistic examination of grammatical difficulty (as defined by the combination of grammatical complexity, learner proficiency, and L1) and type of instruction (input vs. output) could lead to new insights, which allow educators to choose the best educational technique at the right time.

As in the case of CF, research synthesis has provided new insights concerning other forms of FFI. Effects of FFI techniques appear to be influenced by the following characteristics: input-providing, output-prompting, or degree of explicitness. Recent synthesis of 37 experimental studies (19 studies having learners with the Korean L1 and 18 studies having learners with the Persian L1), revealed some parallels to results from the collation of CF studies, with input serving as a type of scaffold for more difficult grammar at lower levels of proficiency (Schenck, 2022a). Studies which included grammatical features of a higher complexity with lower-level learners tended to benefit more from input. This perspective is supported by Modirkhamene et al. (2018), who found that Persian learners at beginner proficiency had higher effect sizes for explicit input *via* Processing Instruction (PI) (*d* = 0.70) and lower effect sizes for output (*d* = 0.52). The finding appears to parallel results found concerning CF. When the past tense is emphasized with beginner learners, input appears to be more effective. In addition to the past *-ed*, other features appear to reveal a relationship between grammatical feature, proficiency, and the benefits of input. In a study by Yang (2008), for example, the present perfect progressive, an inter-phrasal feature, was used with beginning level learners with TOEIC scores of approximately 140–185. Once again, low level learners appear to benefit from input, which may prime the learner, providing essential information to assist with processing and production of a grammatical feature.

Studies that revealed a larger benefit for output-based instruction may also be explained by examining characteristics of the target feature and learner background. In studies of relatively simple, intra-phrasal features like the participial adjective (*bor****ing****/bor****ed***), low intermediate or intermediate learners benefited more from output (Kim, 2002; Yeo, 2002). It appears that low complexity and high proficiency result in larger effects for output, which may push learners to correctly use grammatical features that they are already familiar with. This pattern is repeated in other studies. In a study of high intermediate Korean learners by Jeong and Lee (2018), for example, intra-phrasal features (vocabulary) were emphasized with high school students of “high ability.” In another study of high intermediate and advanced learners by Kim and Nam (2017), more simplistic inter-phrasal collocations like “shake a leg” or “hit the sack” were emphasized. In both cases, output was more effective, further suggesting that low complexity and high proficiency influenced the findings.

Research synthesis of FFI research appears to explain when either input or output-based instruction will be more effective for a specific target feature. As in the case of CF, input associated with other forms of FFI appear most effective when proficiency is low and grammatical difficulty is high. Explicit forms of FFI (PI, explicit grammar instruction, and input enhancement) also tend to have larger effect sizes (Schenck, 2022a), which suggests that the explicit attribute may serve as a scaffold. Overall, results from synthesis of FFI studies may suggest a key link between grammatical difficulty and instruction, which parallels CF (Table 2).

**TABLE 2:** Relationship between grammatical difficulty and instruction.

Type of instruction	Grammatical difficulty	Level of scaffolding
Explicit Input (PI, Explicit Grammar Instruction, Input Enhancement)	Highest Difficulty	Most Highly Scaffolded

Implicit Input (Input Flood)	Intermediate Difficulty	Intermediate Level of Scaffolding

Output (Speaking or Writing Tasks)	Low Difficulty	Low Level of Scaffolding

Review of Table 2 links grammatical difficulty (grammatical complexity, learner proficiency, and L1) with instruction (type of FFI). While insightful, more experimental research is needed. Although research synthesis appears to show relationships between these variables, differences in research methodologies, which ultimately use different treatments and assessments of production, may yield variable results. Experimental studies are required that more holistically explore relationships between variables. In addition, while the effects of different pedagogical techniques that use explicit input (e.g., textual enhancement and PI) appear to be related, little past research has explored such potential relationships, further highlighting a need for additional research. Finally, past studies concerning both CF and other forms of FFI used the same grammatical features repeatedly (e.g., past tense, article, and conditional). By choosing a target feature based upon either intuition or experimental tradition, our understanding of acquisition has remained limited.

## Future Research: Establishing a “Corpus” of Experimental Studies

As revealed by recent synthesis of past research, problems with using the right teaching technique at the right time appears to rest in an incomplete understanding of how grammar is acquired. By looking more holistically at grammatical difficulty (defined collectively as grammatical complexity, learner proficiency, and L1), selection of instructional techniques that maximize acquisition becomes an attainable goal. This also has implications for the design of computer software, which can rapidly modify instruction based upon a learner’s proficiency and L1.

It is important to remember that, while research synthesis provides useful insights, understanding of FFI remains limited. Although some tendencies for FFI effectiveness may be gleaned through collation of past research, studies used for examination contain methodological differences that may lead to variability in results. Thus, a more comprehensive and systematic experimental method is needed to gain a holistic understanding, which can allow computer software to provide emphasis at the right time. Rather than envisioning one research study that incorporates all grammatical features, diverse learners, and instructional style, the collection and collation of a large number of mini-studies may be the answer. Data collection at the individual level may be the key to success. For example, one learner may be presented with a reading and input enhancement for the past tense regular feature. Following a production task, this information can be carefully recorded into a corpus of FFI studies according to the following variables:

1. Amount of input (duration)
2. Kind of input (word count, lexical density, source, etc.)
3. Amount of output (duration)
4. Kind of output (e.g., dictogloss)
5. Target Feature (Complexity level and characteristics)
6. Kind of enhancement (input vs. output-based, degree of explicitness)
7. Proficiency level and L1 of the learner

After meticulously recording characteristics of one learner in a specific circumstance, results can be collated to provide the necessary holistic perspective. In addition to standardization of learner variables, different characteristics of instruction will need to be carefully scrutinized before being recorded into a corpus.

Although reform of research methods is clearly needed, the difficulty involved in creating one multivariate research study is daunting. If multiple variables involved in acquisition can be systematically considered in small-scale studies, they can be collated, thereby providing the holistic perspective needed adapt theory to practice. Computer programs may then be developed to tailor input and output to learner needs.

## Author Contributions

The author confirms being the sole contributor of this work and has approved it for publication.

## Conflict of Interest

The author declares that the research was conducted in the absence of any commercial or financial relationships that could be construed as a potential conflict of interest.

## Publisher’s Note

All claims expressed in this article are solely those of the authors and do not necessarily represent those of their affiliated organizations, or those of the publisher, the editors and the reviewers. Any product that may be evaluated in this article, or claim that may be made by its manufacturer, is not guaranteed or endorsed by the publisher.
